# Can Steam Sterilization Affect the Accuracy of Point-of-Care 3D Printed Polyetheretherketone (PEEK) Customized Cranial Implants? An Investigative Analysis

**DOI:** 10.3390/jcm12072495

**Published:** 2023-03-25

**Authors:** Neha Sharma, Jokin Zubizarreta-Oteiza, Céline Tourbier, Florian M. Thieringer

**Affiliations:** 1 Clinic of Oral and Cranio-Maxillofacial Surgery, University Hospital Basel, 4031 Basel, Switzerland; 2 Medical Additive Manufacturing Research Group (Swiss MAM), Department of Biomedical Engineering, University of Basel, Hegenheimermattweg 167C, 4123 Allschwil, Switzerland

**Keywords:** accuracy, alloplastic implant, computer-assisted, cranioplasty, material extrusion, patient-specific, printing, polymer, sterilization, three-dimensional

## Abstract

Polyetheretherketone (PEEK) has become the biomaterial of choice for repairing craniofacial defects over time. Prospects for the point-of-care (POC) fabrication of PEEK customized implants have surfaced thanks to the developments in three-dimensional (3D) printing systems. Consequently, it has become essential to investigate the characteristics of these in-house fabricated implants so that they meet the necessary standards and eventually provide the intended clinical benefits. This study aimed to investigate the effects of the steam sterilization method on the dimensional accuracy of POC 3D-printed PEEK customized cranial implants. The objective was to assess the influence of standard sterilization procedures on material extrusion-based 3D-printed PEEK customized implants with non-destructive material testing. Fifteen PEEK customized cranial implants were fabricated using an in-house material extrusion-based 3D printer. After fabrication, the cranial implants were digitalized with a professional-grade optical scanner before and after sterilization. The dimensional changes for the 3D-printed PEEK cranial implants were analyzed using medically certified 3D image-based engineering software. The material extrusion 3D-printed PEEK customized cranial implants displayed no statistically significant dimensional difference with steam sterilization (*p* > 0.05). Evaluation of the cranial implants’ accuracy revealed that the dimensions were within the clinically acceptable accuracy level with deviations under 1.00 mm. Steam sterilization does not significantly alter the dimensional accuracy of the in-house 3D-printed PEEK customized cranial implants.

## 1. Introduction

The use of modern three-dimensional (3D) imaging and virtual planning software in craniomaxillofacial surgery has greatly aided in the preoperative planning of surgical interventions [1,2]. When combined with medical additive manufacturing (AM) or three-dimensional (3D) printing, these advancements facilitate the transfer of virtual planning to the surgical site [3,4,5]. Several hospitals worldwide have integrated in-house 3D printing into their daily clinical practices to provide personalized patient care. These studies have discussed point-of-care (POC) 3D printing for various applications, including creating anatomical biomodels for preoperative planning, intraoperative visualization, and pre-bending standard stock implants for simple and complex procedures [6,7,8,9]. In addition, biocompatible 3D printing materials have also made it possible to develop surgical instruments and devices, such as contouring templates, surgical guides, and implant sizing models. These medical devices are used in operating rooms to assist surgeons in performing complex surgeries with more precision [10,11,12].

Recent developmental trends have demonstrated tremendous interest amongst hospitals in the potential use of medical 3D printing in fabricating customized implants tailored to patients’ unique anatomy [13]. Customized implants are typically developed in response to the urgent need for surgeons to manage complex reconstructive cases that necessitate a one-of-a-kind patient-specific approach [14]. The material extrusion-based 3D printing technique is already utilized in hospitals to fabricate anatomical biomodels [15]. Until recently, material extrusion 3D printers could only be used for low-temperature thermoplastics. However, more recent advancements have made it possible to print high-temperature, implantable polymers such as polyetheretherketone (PEEK), opening the way for more sophisticated and customized implant solutions [16,17].

Our previous research has shown the viability of employing material extrusion-based 3D printers designed to produce high-performance, implantable-grade PEEK customized cranial implants with clinically acceptable geometric and morphological characteristics in hospitals [13]. A thermoplastic polymer-based implant is to be sterilized as part of the manufacturing process in the context of implantable medical devices. The International Organization for Standardization (ISO) updates standards for processing healthcare products to provide information to medical device manufacturers [18]. To process a safe medical device for its intended use, it must be sterilized and comply with medical device regulations and international standards [19]. The prerequisite to sterilize an in-house 3D-printed PEEK customized implant raises further concerns about its dimensional accuracy. If a customized implant is deformed during the sterilization procedure, its accuracy will suffer, resulting in a poor clinical outcome.

In healthcare institutions, sterilization is achieved physically or chemically and entails annihilating all microbiological life. Steam sterilization is accomplished by exposing the sterilized items to saturated steam (range: 121–141 °C) under pressure (range: 206–368 kPa) [20]. This technique is also the most often utilized method for sterilizing metallic surgical instruments and medical implants due to its convenience, low cost, and general accessibility in many tertiary-level hospitals [21]. The materials must be stable in high heat and humidity conditions and notably resistant to hydrolytic degradation for autoclave sterilization [22]. When sterilizing polymers, the procedure should be utilized cautiously because heat and steam can drastically deform these materials’ characteristics [23]

With POC 3D printing gaining momentum, it is essential to carefully evaluate the potential effects of sterilization on the biomaterial properties and performance of the implants to ensure that they meet the necessary standards and provide the intended benefits. Therefore, assessing and determining whether and how these in-house 3D-printed implantable biomaterials change morphologically when subjected to high temperatures, pressure, and humidity during the steam sterilization process is imperative. This study assesses and compares the dimensional accuracy of POC material extrusion 3D-printed PEEK customized cranial implants before and after steam sterilization.

## 2. Materials and Methods

### 2.1. Medical Image Processing and Virtual Surgical Planning 

**Figure 1:**
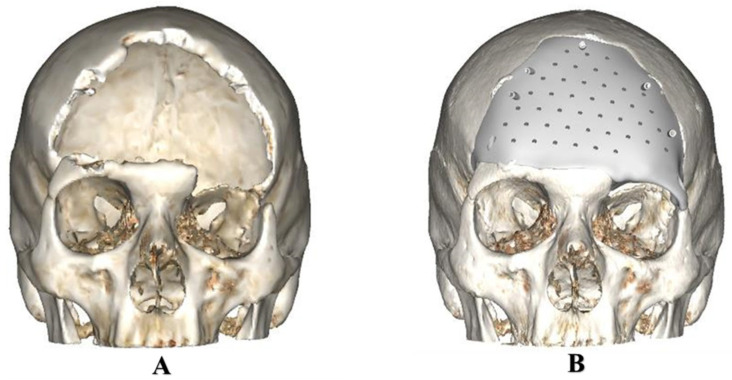
Illustration of a three-dimensional (3D) cranial defect reconstruction with a customized implant. (**A**) Cranial defect model. (**B**) Reconstruction with a customized or patient-specific cranial implant model.

Fifteen anonymized cases with cranial defects were selected from the hospital’s database. The exemplary cases were chosen based on the degree of complexity in computer designing and manufacturing cranial implants [24]. Computed tomography (CT) images were used for image processing in the Digital Imaging and Communications in Medicine (DICOM) format with a slice thickness between 0.75 mm to 1.00 mm. The datasets were imported into an image processing software (MIMICS Innovation Suite v. 25.0, Materialise, Leuven, Belgium). For reconstruction, mirror-based modeling (3-matic medical 17.0, Materialise, Leuven, Belgium) was used in unilateral cranial defects, while normative or previous datasets were used in defects crossing the midline [25]. Figure 1 illustrates an exemplary case of cranial defect reconstruction with a customized implant. The virtually designed cranial implants were exported as standard tessellation language (STL) file format for further processing.

### 2.2. Additive Manufacturing of PEEK Customized Cranial Implants 

**Figure 2:**
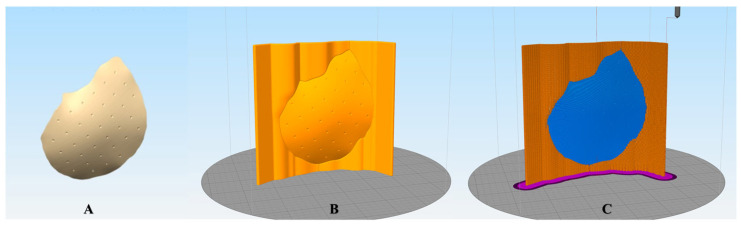
Schematic representation of the pre-processing steps for fabricating polyetheretherketone (PEEK) customized cranial implants. (**A**) Virtually designed customized cranial implant. (**B**) Cranial implant with support structures positioned vertically on the printer’s build platform in the slicing software. (**C**) Toolpath illustration after g-code generation with the selection of defined printing parameters.

The pre-processing steps for fabricating PEEK customized implants included generating support structures and defining printing process parameters. The support structures were modeled per the implants’ orientation on the printer’s build platform. The designed STL files were imported into the 3D printer’s slicing software (Simplify3D 4.1.1, Cincinnati, OH, USA) (Figure 2). Based on the bounding box dimensions, the cranial implants were categorized into three groups (n = 5/group), namely, Group 1: large (90 mm to 150 mm)-, Group 2: medium (50 mm to 90 mm)-, and Group 3: small (35 to 50 mm)-sized implants and the corresponding printing parameters were selected. The respective generated G-code files were then transferred to the 3D printer.

A material extrusion-based 3D printer (Kumovis R1.2, Kumovis GmbH, Munich, Germany) was employed to fabricate the customized implants. All 15 implants were fabricated using a ø1.75 mm medical-grade PEEK filament (Evonik Vestakeep i4 3DF, Evonik Industries AG, Essen, Germany). The material was dried in a forced-air circulation oven (Memmert UF30, Memmert GmbH, Schwabach, Germany) for 12 h at 80 °C prior to each print. Each implant was printed independently and in the center of the build platform. Post-processing procedures were used to manually remove the brim and support structures, followed by drilling the drainage holes.

### 2.3. Digitization of 3D-Printed PEEK Customized Cranial Implants

For the digitization process, an optical white structured-light professional-grade 3D scanner was used (Transcan C, Shining 3D Tech. Co., Ltd., Hangzhou, China). The manufacturer’s specifications were as follows: a camera resolution of 12 megapixels, a scan speed of <70 s (8 scans/turn without texture), a single shot accuracy of 0.05 mm, and a point distance resolution of 37.5 µm. Multi-resolution fusion algorithms were applied, and the implants were digitized in a non-texture, high dynamic range (HDR) under non-watertight settings with the support of an automatic turntable. A scan range of 300 mm × 190 mm was used for digitizing L-size implants, while a scan range of 150 mm × 96 mm was used for M- and S-size implants.

### 2.4. Sterilization Protocol for PEEK Customized Cranial Implants

For the sterilization process, the implants were steam sterilized (autoclave) at 134–137 °C for 18 min, followed by a drying cycle of 30–40 min. Each customized cranial implant was digitized before and after sterilization, and the datasets were exported in an STL file format for analysis.

### 2.5. Dimensional Accuracy Assessment

Deviation analyses were performed over the entire profile of the sterilized 3D printed PEEK customized cranial implants to evaluate the dimensional accuracy. To analyze the congruence of the POC fabricated PEEK customized cranial implants, all post-sterilization optical scans were superimposed onto the pre-sterilized optical scans, which served as the “reference” standard. Automatic alignment was performed using the iterative closest point registration approach (3-matic medical 16.0, Materialise, Leuven, Belgium). By adjusting six-degree (three rotational and three translational) transformation parameters, this technique aligns the two implants by reducing the distance between the two surfaces. To determine the differences between the surfaces, the aligned surfaces were compared. The software’s algorithm matched and automatically determined the differences between the nearest point pairs. The measurements were quantified as mean, median differences (positive and negative variances), standard deviation, root-mean-square error (RMSE), and a color-coded surface distance map. These color-coded heat maps examined the qualitative congruence or incongruence of the pre- and post-sterilized implant surface. The RMSE value was used as the comparative metric to estimate the separation between the two surfaces at anatomically similar regions, served as a measurement indicator of how far the deviations from zero differed between the two datasets, and indicated the overall 3D deviations.

Additionally, the maximum deviation between two surfaces (measured by Hausdorff distance (HD)) was assessed in MeshLab (ISTI-CNR Research Centre, Italy, v2019, https://www.meshlab.net/, accessed on 21 January 2023). The HD denotes the greatest separation between two points of two datasets, both from related mesh sections (i.e., the HD is expected to be equal to 0 in case of a perfect alignment of absolute symmetrical geometries). Theoretically, the value of this parameter varies from 0 to ∞, where 0 denotes that the boundaries of the two regions being compared are identical, while values greater than 0 denote the actual separation between the two surfaces. The values show the surface registration and printed implants’ accuracy after sterilization.

### 2.6. Statistical Analysis

Descriptive statistics, including mean, standard deviation (SD), median, and interquartile ranges (Q1 to Q3), were computed for the average deviations in all the 3D-printed PEEK customized cranial implants. Additionally, RMSE and HD values were calculated for each group to summarize the quantitative characteristics of the dimensional performance. To verify the normality distribution, a Shapiro–Wilk test was conducted. A paired *t*-test was applied to compare the mean measurements of implants before and after sterilization. A difference of less than 0.05 was considered statistically significant. Microsoft Excel 2016 was used to gather and tabulate all data, and the R statistical program (R Core Team, http://www.R-project.org/, accessed on 28 January 2023) was used for statistical analyses.

## 3. Results

The quantitative deviation analysis depicting the average distance between pre- and post-sterilized cranial implants within each group is summarized in Table 1. The composite analysis for all the fifteen post-sterilized 3D-printed PEEK cranial implants had a mean RMSE (SD) value of 0.16 (0.09) mm and a median (Q1 to Q3) RMSE value of 0.13 (0.09 to 0.21) mm.

**Figure 3:**
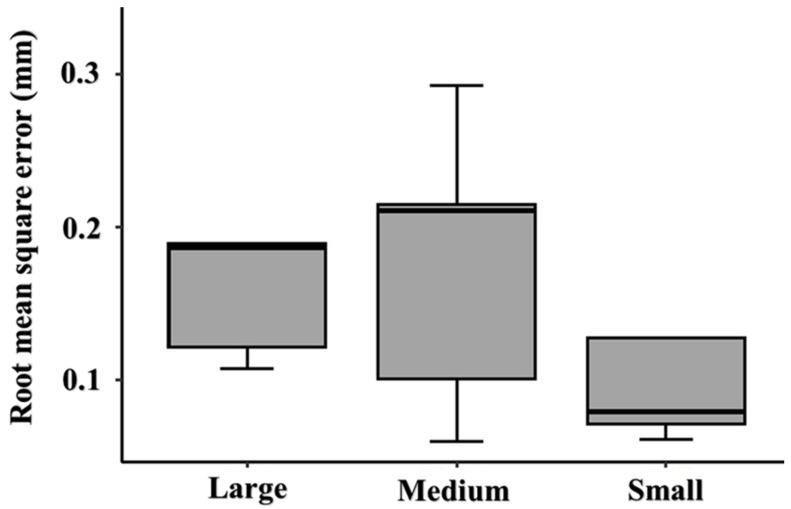
Box plot representing comparative analysis of average deviation values within each group.

Figure 3 illustrates a comparative analysis of the quantitative assessment of dimensional changes within each group of material extrusion 3D-printed PEEK customized cranial implants. The implants displayed no statistically significant difference with sterilization (*p* > 0.05).

**Figure 4:**
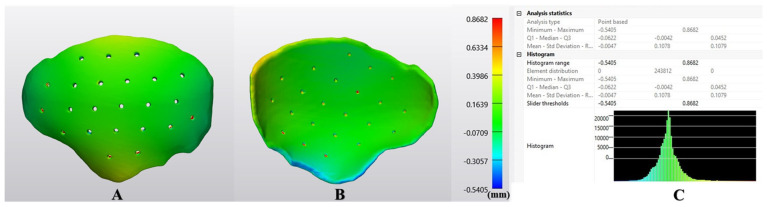
Heatmap illustrating the deviations (mm) in a post-sterilized large-sized 3D-printed PEEK customized cranial implant. Visualization of (**A**) outer (squamous) surface; (**B**) inner (cerebral) surface; (**C**) descriptive analysis.

Figure 4 illustrates the deviations in an exemplary large-sized cranial implant in a color-coded map. The shaded area in the red zone indicates that the post-sterilized implant’s region is more than the reference’s, indicating that the deviation value is positive. The shaded area in the blue zone denotes a lower post-sterilized implant area than the reference, indicating a negative deviation value. The green zone represents the overlap area between the pre-and post-sterilized implant models. As noticed in Figure 4, most areas are green, indicating that the post-sterilized implant’s size is comparable to the reference model. Furthermore, a few areas around the edge of the implant exhibited a slight negative incongruence from the reference models.

**Figure 5:**
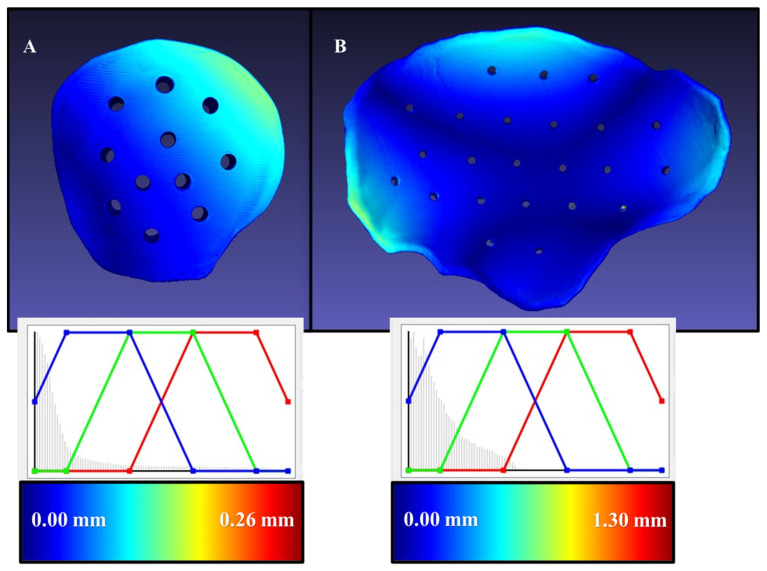
Heatmaps depicting the Hausdorff distances for two exemplary 3D printed PEEK customized cranial implants. The minimal and maximal values are color-coded and displayed in millimeters (mm). (**A**) Small-sized cranial implant model; (**B**) large-sized cranial implant model.

The arithmetic mean of Hausdorff’s maximum distances for the sterilized 3D-printed PEEK customized implants was 0.96 mm (SD 0.54) and ranged from 0.15 to 1.30 mm. The exemplary models with overlayed heatmaps of Hausdorff metrics are presented in Figure 5 and represent the minimal and maximal values. The blue region represents a perfect overlap between the two surfaces, and the regions closer to the red spectrum represent increased deviations. The equalizer in the representative graphs represents the data distribution of individual points across the implant’s entire profile. It can be seen that the representative small-size cranial implant had a Hausdorff’s maximum distance value of 0.26 mm, while the large-sized cranial implant had a Hausdorff’s maximum distance of 1.30 mm, with most of the dataset distribution closer to the blue region. These results concluded that steam sterilization had no clinically significant deviations in the fabricated PEEK customized cranial implants.

**Figure 6:**
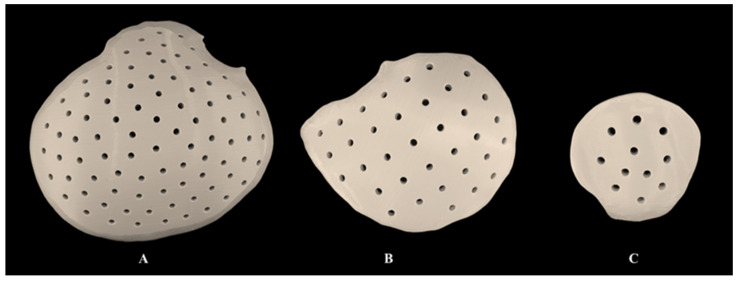
Steam sterilized, material extrusion-based 3D-printed polyetheretherketone (PEEK) customized cranial implants. (**A**) Large-sized; (**B**) medium-sized; (**C**) small-sized.

On visual inspection, the subjective assessment revealed no signs of discoloration or structural discrepancies in the sterilized material extrusion-based 3D-printed PEEK customized cranial implants (Figure 6). Furthermore, the implant’s marginal fit was assessed subjectively on 3D-printed cranial defect anatomical models by a craniomaxillofacial surgeon and was considered clinically acceptable.

## 4. Discussion

Current developments in digital technology have altered how modern craniomaxillofacial surgeons use patient data for individualized care [2,3,7]. PEEK has become the preferred biomaterial to repair craniofacial deformities over time [26,27]. With the advancements in 3D printing technologies, possibilities for the POC manufacturing of PEEK customized implants have emerged; however, little is known about material extrusion 3D-printed PEEK implants’ clinical translation. Despite the long history of milled PEEK implants in cranial reconstructions, the development of commercially available medical-grade PEEK filaments for material extrusion-based 3D printing is still relatively new [13]. All novel technologies are exploratory by definition, hence guidelines and definitive quality control measures are required to use these cutting-edge methods in order to mitigate the potential risks [4]. To ensure that the in-house 3D-printed customized implants meet medical device requirements and deliver the desired results, it is critical to evaluate all potential effects of sterilization on the biomaterial properties and functionality of these implants. As cranial reconstructions are invasive surgical procedures, implant contamination with microorganisms may result in infection and cause a poor outcome [28,29]. The current study investigated the influence of steam heat (autoclave) sterilization on the dimensional characteristics of POC material extrusion-based 3D-printed PEEK customized cranial implants. Our findings revealed that the material extrusion-based 3D-printed PEEK cranial implants were within the acceptable dimensional accuracy range for cranioplasty reconstructive surgeries post-sterilization, with deviations under 1.0 mm. Furthermore, steam sterilization had no statistically significant influence on the dimensional characteristics of the 3D-printed PEEK implants.

With advancements in 3D printing technology, the prospects for bespoke PEEK surgical implants are being developed at the POC, but little is known about their potential clinical utility [30,31,32]. Many factors, including the steps of virtual surgical planning, fabrication, and post-processing, determine the accuracy of a customized implant [33,34]. The accurate transfer of virtual surgical planning to the actual clinical situation and structural stability of the customized implant after additional processing steps, such as steam sterilization, are critical in ensuring a biomaterial’s suitability for patient use [35,36]. Our preliminary findings show a color-coded surface-deviation map based on the RMSE, illustrating point-based and overall conformance distance deviations. The co-registered pre- and post-sterilized customized PEEK cranial implants displayed overall average deviations under 0.5 mm across the implant’s surface areas. The HD-based analysis revealed that most implants’ maximum deviations were under 1.00 mm. The primary value of these analyses is the quantitative confirmation of our preclinical experience that the in-house fabricated and sterilized PEEK customized cranial implants provide a close morphologic semblance to the pre-sterilized counterparts.

Color-coded distance maps are an analytical tool in most computer-aided design software used to calculate the relative distance or deviation between two 3D surface meshes [37]. Previous studies have used the color mapping method to assess the dimensional accuracy of 3D-printed PEEK cranial implants [13,31]. Generally, green indicates zero deviation, indicating a suitable congruence between the two 3D model surface meshes (planned vs. actual). Green, yellow, and blue colors predominated on most surface of the implant, with fewer red colors seen in the regions of the drainage holes. Despite a minor color variability, the post-sterilized implant segments that correlated more closely with the pre-sterilized counterparts had less variation and intensity of color, indicating that the implants retain their morphological shape and contour after steam sterilization precisely. However, slight color variability was noticeable at the margins, indicating that thinner implants can undergo deformation during sterilization. Therefore, a criterion of the minimum thickness of implants is imperative in material extrusion 3D-printed PEEK implants.

Furthermore, erroneous scan data and limitations of the registration protocol can also cause some deviations that can be seen in the final analysis. A less than 2 mm difference between the planned and actual surfaces has been considered clinically acceptable in virtual surgical planning [13,37]. In the present study, the results rejected the null hypothesis that all values of mean deviations would be higher than 2 mm. Although this method accurately evaluated the effect of sterilization on the implant regions, an implant may be considered unacceptable if it deforms at the margins and thereby would have limited clinical applicability. Therefore, a subjective assessment of the implants is essential to ascertain that the post-sterilized implants have a clinically acceptable marginal fit.

In clinical research, the importance of the post-processing steps affecting an implant’s dimensional conformity is frequently understated. In a pilot study, Dautzenberg et al. [38] investigated the effects of steam sterilization on material extrusion-based 3D printed polymeric materials and concluded that most materials exhibited more significant morphological variations in steam-pressure sterilization. However, it should be noted that the tested materials in their study were thermoplastics with low melting point temperatures, such as polylactic acid (PLA), polycarbonates (PC), and acrylonitrile butadiene styrene (ABS). The glass transition temperature (Tg) of polymers predicts how heat-induced sterilization causes chemical and structural changes in polymer properties [39]. A material with Tg close to steam-sterilization temperature can affect the polymeric chain mobility and its dimensional and mechanical characteristics [40].

PEEK is a semi-crystalline polymer whose Tg is 145 °C and melting temperature, Tm, is 343 °C [41]. Our investigation found no discernible difference in the mean deviation across the implant groups. Such outcomes could be explained by the fact that 134 °C is a relatively low sterilization temperature compared to PEEK’s thermal degradation temperature (575 °C and 580 °C) [42]. With a melting point of 343 °C, the material extrusion 3D-printed PEEK customized cranial implants displayed thermal degradation resistance unaffecting the dimensional characteristics [43]. This in vitro study has certain limitations. For this study, we followed a sterilization protocol frequently employed in the sterilization department of the University hospital. The current study did not examine the impact of alternate protocols with longer or shorter cycles, higher or lower temperatures, or different pressure settings.

Until now, PEEK customized implants have been manufactured by external med-tech companies. This production method can take several weeks and necessitates numerous meetings between surgeons and biomedical engineers. Furthermore, the costs of these customized PEEK cranial implants are high (ranging from 3000 to 10,000 €) and vary depending on the size and complexity of the defect [33]. On the other hand, POC manufacturing of PEEK implants could be highly beneficial, thereby reducing the production lead times and treatment times significantly. The adoption of customized implants will be accelerated by the financial savings brought about by using filament-based fabrication for PEEK cranial plates compared to conventional technologies. For instance, the procurement of a PEEK milling machine or powder bed technology-based 3D printing is often more expensive regarding the original hardware investments and the subsequent post-processing requirements.

Furthermore, hospitals considering embarking on POC manufacturing must use certified equipment and adhere to the regulatory framework. Currently, the described POC manufacturing process of 3D printed PEEK patient-matched cranial implants is exempt under Article 5(5) of Regulations (EU) 2017/745 (Medical Devices Regulation, MDR). However, under this exemption, healthcare institutions must still provide information on the manufacture and use of in-house medical devices to their competent healthcare authority upon request, including a justification for the design envelope, process parameters, manufacturing, risk analysis, and modifications (when applicable). Translating this workflow is feasible for other institutions, provided each institute declares that the patient-matched implants are only manufactured and used in the respective health institution and meet the applicable general safety and performance requirements (GSPR) of the medical devices regulation (EU 2017/745).

## 5. Conclusions

To the knowledge of the authors, this is the first study to assess the sterilization effects on the dimensional accuracy of POC-manufactured PEEK cranial implants. The personalized 3D-printed PEEK cranial implants displayed high dimensional accuracy concerning the geometric characteristics. To deploy 3D-printed implants regularly in clinical practice in operating rooms, we hypothesize that hospitals embarking on the concept of POC manufacturing of customized implants need to set up their quality control and quality management systems, thereby establishing a validated and regulated workflow for the entire production cycle.

## Figures and Tables

**Table 1:** Average deviation values (in mm) for each group.

Implant Groups	Mean RMSE ± SD	Median RMSE (Q1 to Q3)
Large-sized	0.19 ± 0.09	0.19 (0.12 to 0.19)
Medium-sized	0.18 ± 0.09	0.21 (0.10 to 0.22)
Small-sized	0.13 ± 0.10	0.08 (0.07 to 0.13)
Overall	0.16 ± 0.09	0.13 (0.09 to 0.21)

SD—standard deviation; RMSE—root-mean-square error.

## Data Availability

The original contributions presented in the study are included in the article; further inquiries can be directed to the corresponding author.
